# Light stress elicits soilborne disease suppression mediated by root-secreted flavonoids in *Panax notoginseng*

**DOI:** 10.1093/hr/uhae213

**Published:** 2024-07-30

**Authors:** Haiyan Fang, Cunwu Guo, Xinyue Mei, Minwen Hao, Jiayin Zhang, Lifen Luo, Haijiao Liu, Yixiang Liu, Huichuan Huang, Xiahong He, Youyong Zhu, Min Yang, Shusheng Zhu

**Affiliations:** State Key Laboratory for Conservation and Utilization of Bio-Resources in Yunnan, Yunnan Agricultural University, Kunming, 650201, China; State Key Laboratory for Conservation and Utilization of Bio-Resources in Yunnan, Yunnan Agricultural University, Kunming, 650201, China; State Key Laboratory for Conservation and Utilization of Bio-Resources in Yunnan, Yunnan Agricultural University, Kunming, 650201, China; State Key Laboratory for Conservation and Utilization of Bio-Resources in Yunnan, Yunnan Agricultural University, Kunming, 650201, China; Yuanjiang County Tobacco Monopoly Bureau, Yuxi, 653399, China; State Key Laboratory for Conservation and Utilization of Bio-Resources in Yunnan, Yunnan Agricultural University, Kunming, 650201, China; State Key Laboratory for Conservation and Utilization of Bio-Resources in Yunnan, Yunnan Agricultural University, Kunming, 650201, China; State Key Laboratory for Conservation and Utilization of Bio-Resources in Yunnan, Yunnan Agricultural University, Kunming, 650201, China; State Key Laboratory for Conservation and Utilization of Bio-Resources in Yunnan, Yunnan Agricultural University, Kunming, 650201, China; Key Laboratory for Agro-Biodiversity and Pest Control of Ministry of Education, Yunnan Agricultural University, Kunming, 650201, China; State Key Laboratory for Conservation and Utilization of Bio-Resources in Yunnan, Yunnan Agricultural University, Kunming, 650201, China; Key Laboratory for Agro-Biodiversity and Pest Control of Ministry of Education, Yunnan Agricultural University, Kunming, 650201, China; State Key Laboratory for Conservation and Utilization of Bio-Resources in Yunnan, Yunnan Agricultural University, Kunming, 650201, China; Key Laboratory for Agro-Biodiversity and Pest Control of Ministry of Education, Yunnan Agricultural University, Kunming, 650201, China; State Key Laboratory for Conservation and Utilization of Bio-Resources in Yunnan, Yunnan Agricultural University, Kunming, 650201, China; Key Laboratory for Agro-Biodiversity and Pest Control of Ministry of Education, Yunnan Agricultural University, Kunming, 650201, China; State Key Laboratory for Conservation and Utilization of Bio-Resources in Yunnan, Yunnan Agricultural University, Kunming, 650201, China; Key Laboratory for Agro-Biodiversity and Pest Control of Ministry of Education, Yunnan Agricultural University, Kunming, 650201, China; State Key Laboratory for Conservation and Utilization of Bio-Resources in Yunnan, Yunnan Agricultural University, Kunming, 650201, China; Key Laboratory for Agro-Biodiversity and Pest Control of Ministry of Education, Yunnan Agricultural University, Kunming, 650201, China

## Abstract

Developing disease-suppressive soils is an effective approach for managing soilborne diseases, which can be achieved through crop metabolism and root secretion modification to recruit beneficial soil microbiota. Many factors, such as light, can elicit and modify plant metabolomic activities, resulting in disease suppression. To investigate the impact of light, *Panax notoginseng* was planted in a greenhouse and forest, conditioned with three levels of light intensities, including the optimal (15% light transmittance of full light), suboptimal low (5% light transmittance of full light) and suboptimal high (30% light transmittance of full light) intensities. We assessed the rhizosphere microbiota of *P. notoginseng* and root rot disease caused by soilborne pathogen *Ilyonectria destructans*, and elucidated the mechanism. Results showed that suboptimal light conditions alleviated root rot disease of *P. notoginseng* by enriching beneficial microbiota in the rhizosphere. Both low and high light stresses enhanced the secondary metabolism profile in favor of plant defense, particularly the flavonoid pathway. Notably, high light stress demonstrated a robust ability to promote flavonoid metabolism and secretion, resulting in the enrichment of more beneficial microorganisms that suppressed the soilborne pathogen *I. destructans*. These findings highlight the potential for adjusting canopy light intensities to improve soil health and promote sustainable agriculture.

## Introduction

Soilborne diseases are a limiting factor to crop health [1]. Managing these diseases is challenging due to factors like complex infections from multiple pathogens, a wide range of host plants that harbor pathogens, and long-term survival of pathogens in the soil [2, 3]. Various strategies have been employed to mitigate soilborne diseases, such as crop rotation, fungicide application, soil sterilization, and using plant growth-promoting rhizobacteria (PGPRs) [4, 5].

However, each of these approaches has some limitations. For example, the use of chemical pesticides has resulted in environmental pollution [6], while PGPRs often struggle with effectiveness due to soil complexity [7]. Enhancing plant metabolism and root exudate secretion to recruit beneficial microbes presents a promising strategy to establish a defensive rhizosphere against soilborne diseases.

The composition of the rhizosphere microbiome is influenced by both host plant varieties and environmental factors [8, 9]. Previous studies, including our own, have demonstrated that plants can modify root metabolism and secretion to recruit beneficial microbes in response to biotic and abiotic stresses, a mechanism known as the ‘cry for help’ strategy [10, 11]. This capability can lead to disease-suppressive soils as plants stimulated by stresses enhance beneficial microbial communities [12–14]. Additionally, aboveground plant parts stimulated by whiteflies can suppress soilborne pathogens such as *Agrobacterium tumefaciens* and *Ralstonia solanacearum* in plant roots through systemic signal transduction [15]. Therefore, an innovative strategy to alleviate diseases in plants is the recruitment of beneficial microbes in the rhizosphere through the elicitation effects in aboveground tissues. However, the application of such measures through pathogen or insect stimulation is limited in conventional cultural practices due to their uncontrollability.

**Figure 1:**
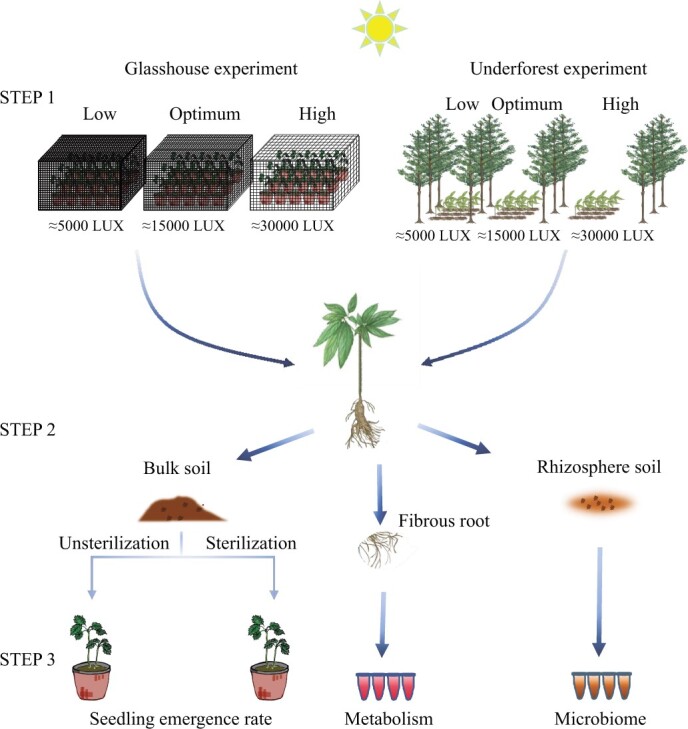
Flowchart of experiments in glasshouse and underforest conditions, examining the impact of light intensity on disease suppression. Step 1, three light intensity treatments, ranging from low to high. Step 2, sampling rhizosphere soil, bulk soil, and fibrous roots. Step 3, bulk soil, either unsterilized or sterilized, was used to evaluate subsequent seedling emergence of *P. notoginseng*. The rhizosphere soil was used for analyzing the soil microbiome. The fibrous roots were used for metabolism analysis.

Abiotic stresses, such as light, temperature, and soil moisture, influence the rhizosphere microbiome by eliciting and modifying plant root metabolisms and secretions [16, 17]. Plants release a substantial portion of their photosynthetic products into the soil and these products impact soil microorganisms [18, 19]. Light can influence the rhizosphere microbiome through a shoot–root–microbiota pathway, where suboptimal light conditions increase beneficial microorganisms in the rhizosphere, thereby promoting plant growth [20]. Furthermore, the growth and metabolism of plants are influenced by constant fluctuations of natural light [21]. The intensity and spectral characteristics of light significantly impact how plants adapt their primary and secondary metabolism to environmental changes [22, 23]. Therefore, adjusting the light intensity in the crop canopy is an effective measure for regulating plant growth, quality, and health in agricultural production [24, 25]. However, due to the complex spectral characteristics, it is challenging to optimize indoor plant growth conditions through spectrum adjustment [26]. Nevertheless, it remains unclear whether changes in light intensity can recruit native beneficial microbiota by altering root metabolisms to suppress soilborne diseases.

*Panax notoginseng*, a medicinal plant belonging to the Araliaceae family, thrives best in shaded environments [27]. However, it is prone to soilborne diseases [28]. To maintain the health of *P. notoginseng* in the field, it is essential to manage light exposure through shading treatments or adjusting light intensity at different growth stages [29]. Successful cultivation of *P. notoginseng* under forest conditions with naturally fluctuating light has shown reduced disease incidence and improved product quality [30]. This makes *P. notoginseng* an ideal model for studying the effects of light on suppressing soilborne diseases and investigating how light stress affects root exudates and the recruitment of beneficial microbes.

## Results

### Light stress modifies soil microbiome for alleviating *P. notoginseng* root rot

**Figure 2:**
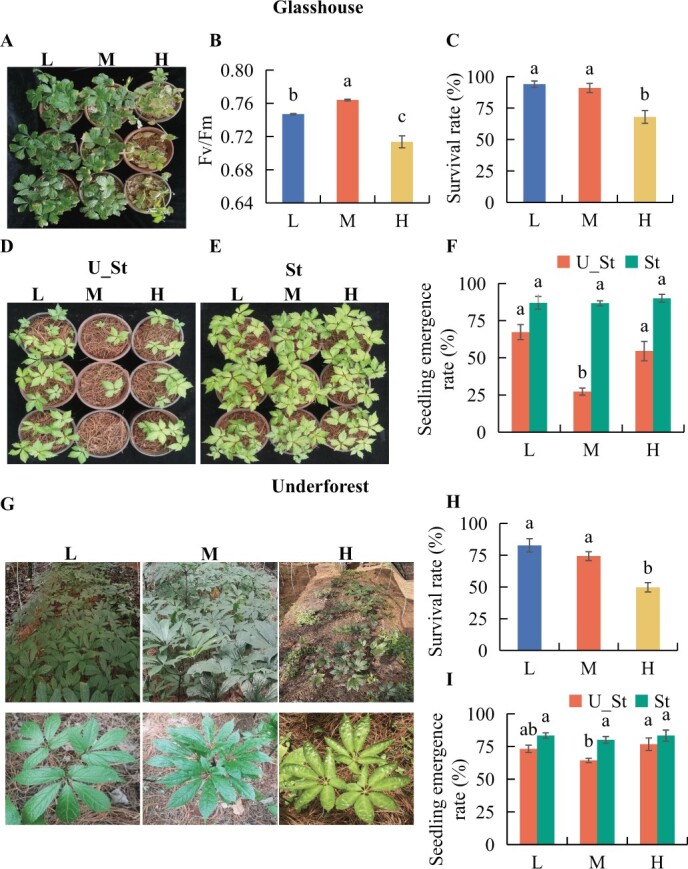
Effects of light intensity on growth and subsequent seedling emergence of *P. notoginseng*. **A** Glasshouse observation. **B** Maximum efficiency (*F*_v_/*F*_m_) of photosystem II in leaves. **C** Seedling survival rate. **D**–**F** visualization of growth (**D**, **E**) and emergence (**F**) for *P. notoginseng* grown in bulk soil collected from the glasshouse experiment, treated with sterilization (St) and without sterilization (U_St). **G**, **H** Growth visualization (**G**) and seedling survival (**H**) of *P. notoginseng* in the underforest experiment. **I** Seedling emergence of subsequent *P. notoginseng* in bulk soil collected from the underforest experiment, treated with sterilization (St) and without sterilization (U_St). The data points represent means ± standard error. Different letters indicate significant differences among treatments (*P* < 0.05).

Glasshouse experiments (Fig. 1) showed that high light intensity resulted in decreased survival rates (Fig. 2A) and the *F*_v_/*F*_m_ index (Fig. 2B) of *P. notoginseng* was significantly reduced under low or high light intensity treatments compared with the condition of optimum light (Fig. 2A and C). However, subsequent cropping experiments revealed that the emergence rates of plants were significantly higher in soil conditioned with low and high light intensities compared with soil with moderate light treatment (Fig. 2D and F). Notably, these differences vanished upon soil sterilization (Fig. 2E and F). The results from underforest experiments (Fig. 1) were consistent with those from the glasshouse; the survival rates of *P. notoginseng* gradually decreased with increasing light intensity (Fig. 2G and H). Additionally, the seedling emergence rates of subsequent *P. notoginseng* cultivation significantly increased under high light intensity compared with the optimum light treatment, but these differences also disappeared after sterilization (Fig. 2I).

**Figure 3:**
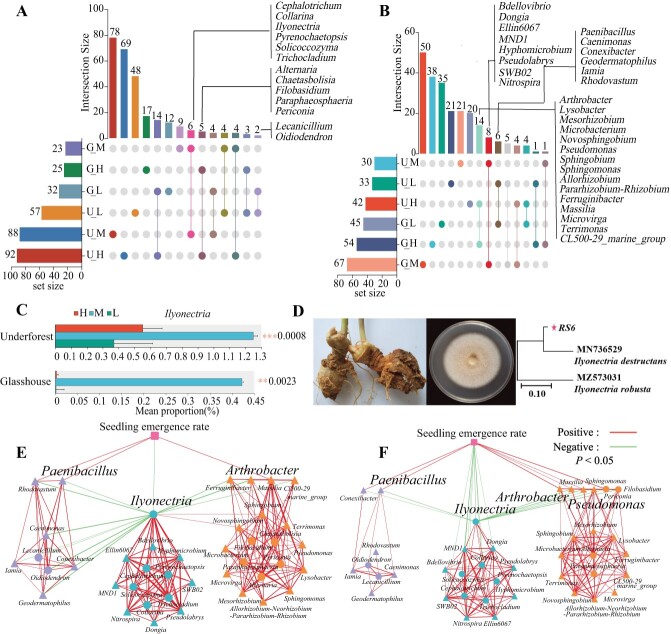
Effects of light intensities on rhizosphere soil of *P. notoginseng* through modifications of co-regulated marker microorganisms in both the glasshouse and underforest experiments. **A**, **B** Co-regulated fungal (**A**) and bacterial (**B**) marker genera identified in both glasshouse and underforest experiments. **C** Relative abundance of *Ilyonectria* in rhizosphere soil of *P. notoginseng*. **D**  *Ilyonectria destructans* strain RS6 isolated from root rot tissues of *P. notoginseng*. **E**, **F** Networks illustrating the relationships between fungal and bacterial marker genera, seedling emergence, and pathogenic *Ilyonectria* genera across the underforest (**E**) and glasshouse (**F**) experiments.

### Light stress enriches antagonistic organisms of rhizosphere microbiome against soilborne pathogens

To determine the effects of light intensity on the soil microbiome and subsequent suppression of soilborne diseases, rhizosphere soils from *P. notoginseng* in underforest and glasshouse experiments were analyzed (Fig. 1). Principal coordinate analysis (PCoA) revealed significant differences in microbial community composition among the three light treatments in both glasshouse and underforest experiments (Supplementary Data Fig. S1A–D).

In the underforest experiment, 776 fungal genera and 897 bacterial genera were identified. Among these taxa, 366 fungal genera (Supplementary Data Table S1) and 332 bacterial genera (Supplementary Data Table S2) were significantly influenced by light intensity, as determined by the Kruskal–Wallis *H* test (*P* < 0.05). Subsequently, biomarker genera were identified using LEfSe analysis. A total of 235 fungi (Supplementary Data Table S3) and 104 bacteria (Supplementary Data Table S4) were identified as biomarkers. In the glasshouse experiment, a total of 594 fungal genera and 433 bacterial genera were found. Among them, 117 fungal genera (Supplementary Data Table S5) and 282 bacterial genera (Supplementary Data Table S6) were significantly regulated by light intensity. In addition, 82 fungi (Supplementary Data Table S7) and 168 bacteria (Supplementary Data Table S8) were identified as biomarkers.

In both underforest and glasshouse experiments, further examination revealed co-regulated biomarker genera exhibiting similar patterns under the same light-intensity treatments (Fig. 3A and B; Supplementary Data Tables S9 and S10). Specifically, there were 2, 6, and 5 co-regulated fungal biomarkers, along with 6, 8, and 14 co-regulated bacterial biomarkers in the low, middle, and high light-intensity treatments, respectively (Fig. 3A and B). Notably, the pathogenic *Ilyonectria* was co-regulated under the optimum light treatment (Fig. 3A). The relative abundance of *Ilyonectria* significantly decreased under low and high light-intensity treatments (Fig. 3C).

The RS6 strain, isolated from *P. notoginseng* showing root rot, was identified as *I. destructans* (Fig. 3D). Correlation analysis demonstrated that most co-regulated microorganisms under low or high light intensities exhibited a negative correlation with *Ilyonectria* but a positive correlation with seedling emergence rates (Fig. 3E and F). However, microorganisms under the optimum light intensity showed a negative correlation with seedling emergence rates (Fig. 3E and F). Among these genera, *Paenibacillus* was co-regulated by low light, while *Arthrobacter*, *Massilia*, and *Filobasidium* were co-regulated by high light (Fig. 3E and F).

**Figure 4:**
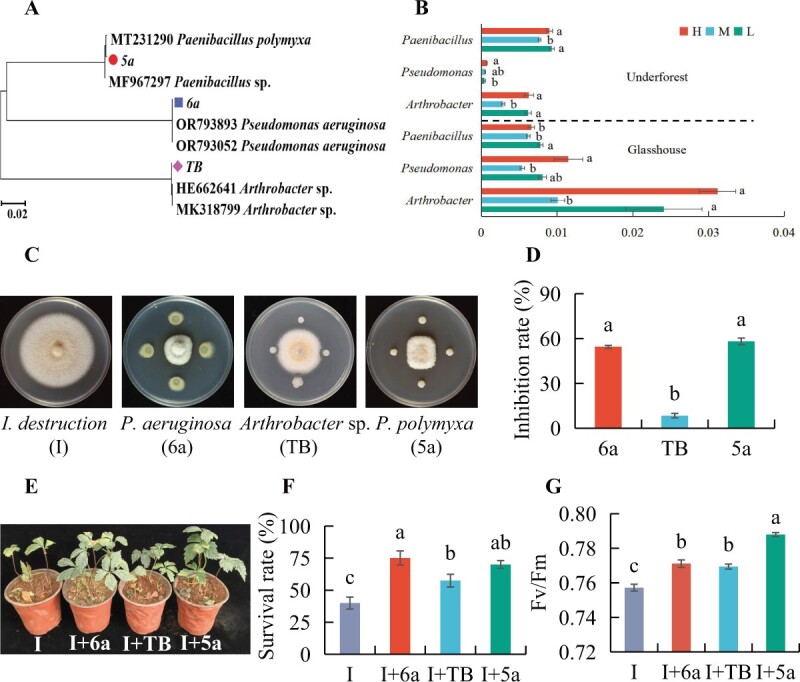
Effects of marker bacteria in inhibiting *I. destructans* and promoting the health of *P. notoginseng*. **A**, **B** Phylogenetic tree displaying the relationship among the marker bacteria (**A**) and relative abundances of *Paenibacillus*, *Arthrobacter*, and *Pseudomonas* in the soil (**B**), as observed in both the glasshouse and underforest experiments. **C**, **D** Antagonistic activity (**C**) and inhibition rate (**D**) of marker bacteria against *I. destructans*. **E**–**G** Effects of marker bacteria (6a, TB, and 5a) on *I. destructans* in soil (**E**), survival (**F**), and maximum efficiency of photosystem II (*F*_v_/*F*_m_) (**G**) of *P. notoginseng*. Mean values ± standard errors are represented. Different letters on the bars denote significant differences among treatments (*P* < 0.05).

To confirm these correlations, three culturable strains, including *Arthrobacter* sp. (TB), *Pseudomonas aeruginosa* (6a), and *Paenibacillus polymyxa* (5a), were isolated from the rhizosphere soil and subsequently identified (Fig. 4A). These genera exhibited significant enrichment under low and high light stress conditions compared with the middle light condition (Fig. 4B). *Pseudomonas aeruginosa* (6a), *Arthrobacter* sp. (TB), and *P. polymyxa* (5a) exhibited antagonistic activity against *I. destructans* (Fig. 4C and D). Subsequently, soil inoculated with *I. destructans* was supplemented with *P. aeruginosa* (6a), *Arthrobacter* sp. (TB), and *P. polymyxa* (5a) to assess their effects on the growth of *P. notoginseng*. The results demonstrated that the survival of seeds and the *F*_v_/*F*_m_ of leaves in soil inoculated with *P. aeruginosa* (6a), *Arthrobacter* sp. (TB), and *P. polymyxa* (5a) were significantly increased compared with the control group, which was solely inoculated with *I. destructans* (Fig. 4E–G).

### Effects of light intensity on metabolite profile of *P. notoginseng* fibrous roots

**Figure 5:**
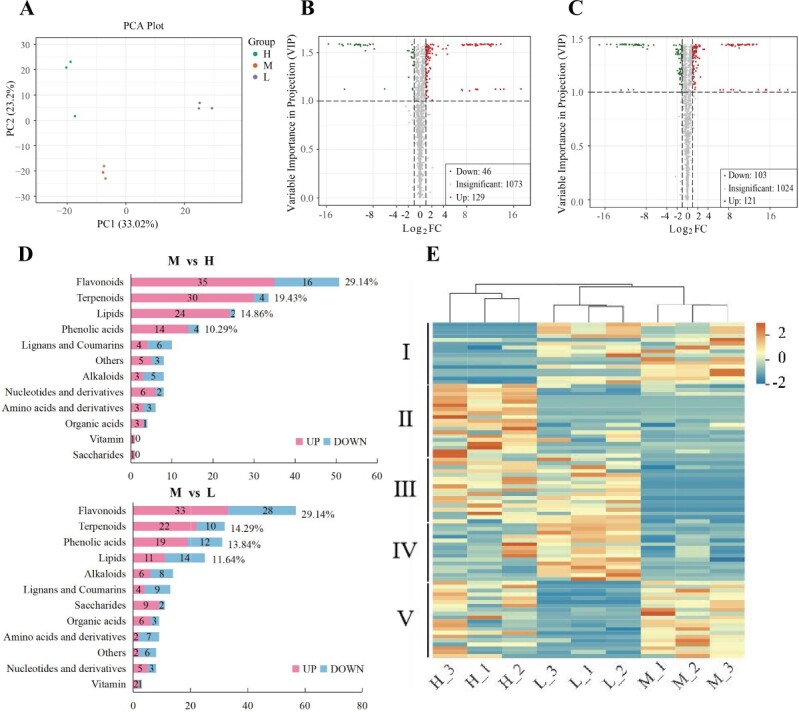
Metabolite profile of *P. notoginseng* fibrous roots treated with different light intensities. **A** PCA of metabolite profiles. **B**, **C** Volcano plots comparing metabolite changes under high (**B**) and low (**C**) light intensities relative to the optimal light condition. **D** Assessment of metabolite categories under high (M versus H) and low (M versus L) light intensities, compared with the optimal light condition. **E** Heat map illustrating the differential expression of flavonoids.

A total of 1304 metabolites were detected in the fibrous roots of *P. notoginseng*, which were categorized into 10 distinct classes. These classes included 238 terpenes, 195 flavonoids, 179 lipids, 174 phenolic acids, 92 alkaloids, 98 amino acids and their derivatives, 76 organic acids, 64 nucleotides and their derivatives, 51 lignins and coumarins, and 137 from other classes (Supplementary Data Table S11). Principal component analysis (PCA) demonstrated distinct differentiation among the three treatments (Fig. 5A). The high and low light treatments exhibited significant changes in metabolite profiles, with 175 (Fig. 5B) and 224 (Fig. 5C) unique metabolites identified, respectively, when compared with the optimal light condition. Notably, the flavonoid class displayed the highest proportion of differentially expressed metabolites, followed by terpenoids, lipids, and phenolic acids in both the high and low light treatments (Fig. 5D). The expression profiles of flavonoids were divided into five groups under the three different light conditions. Specifically, Group II metabolites were significantly upregulated under high light intensity, Group IV under low light intensity, and Group III under both low and high light treatments (Fig. 5E).

### Flavonoid metabolites suppress pathogens but promote antagonistic bacteria

**Figure 6:**
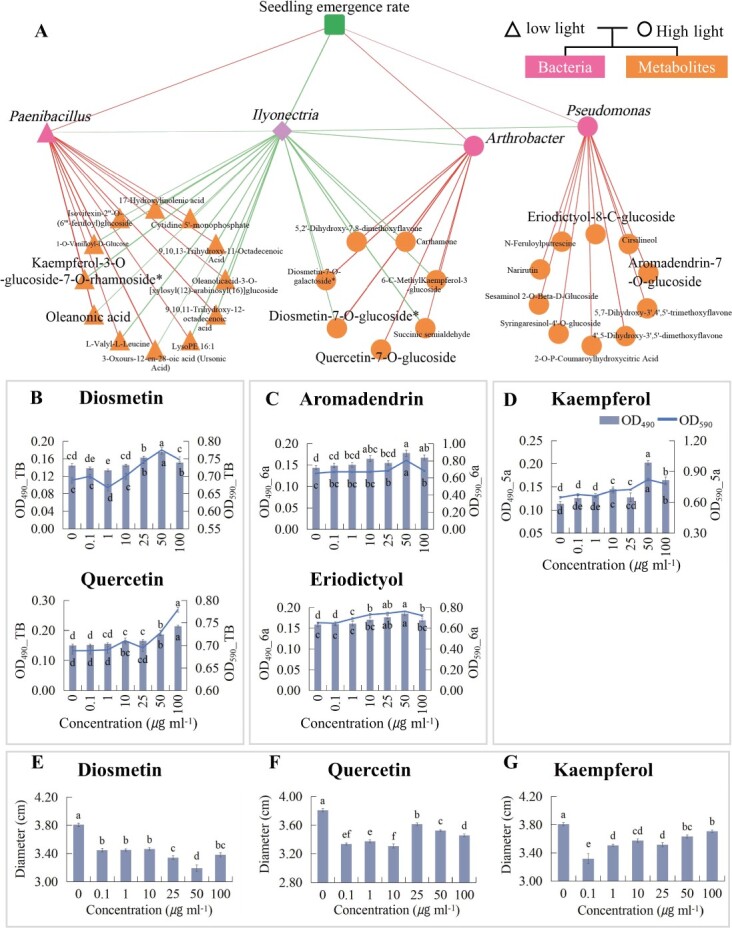
Flavonoid metabolites derived from *P. notoginseng* suppress the pathogen *I. destructans* while promoting the growth of its antagonistic bacteria. **A** Interplay between differential metabolites and *I. destructans*, as well as its antagonistic bacteria. **B** Impact of diosmetin and quercetin on biofilm formation and growth of *Arthrobacter* sp. (TB) strain. **C** Effects of aromadendrin and eriodictyol on biofilm formation and growth of *Pseudomonas aeruginosa* (6a). **D** Influence of kaempferol on biofilm formation and growth of *Paenibacillus polymyxa* (5a). **E**–**G** Suppressing effect of diosmetin (**E**), quercetin (**F**), and kaempferol (**G**) on growth of *I. destructans*. Mean values ± standard errors are represented. Significantly different treatments are indicated by distinct letters on the bars (*P* < 0.05).

A correlation analysis was conducted to examine the relationship between differential metabolites and the presence of *Ilyonectria*, as well as its antagonistic bacteria *Pseudomonas*, *Arthrobacter*, and *Paenibacillus* (Fig. 6A). Under high light-intensity treatment, the majority of correlated differential metabolites were classified as flavonoids. Notably, compounds that included 5,2′-dihydroxy-7,8-dimethoxyflavone, 6-*C*-methylkaempferol-3-glucoside, carthamone, diosmetin-7-*O*-galactoside, diosmetin-7-*O*-glucoside, and quercetin-7-*O*-glucoside exhibited a significant negative correlation with the relative abundance of *Ilyonectria*, but a positive correlation with the relative abundance of *Arthrobacter*. Other flavonoids, including eriodictyol-8-*C*-glucoside, aromadendrin-7-*O*-glucoside, 4′,5-dihydroxy-3′,5′- dimethoxyflavone, naringenin-7-*O*-rutinoside (narirutin), and cirsilineol, displayed a significant positive correlation with the relative abundance of *Pseudomonas*. In the low light treatment, the content of kaempferol-3-*O*-glucoside-7-*O*-rhamnoside, isovitexin-2″-*O*-(6″’-feruloyl) glucoside, 3-oxoolean-12-en-28-oic acid (oleanonic acid), and 3-oxo-urs-12-en-28-oic acid (ursonic acid) exhibited a significant negative correlation with *Ilyonectria*, but a positive correlation with *Paenibacillus*.

To validate these relationships, corresponding aglycones were selected for further testing, given the inherent instability of glycosylated flavonoids in the soil environment. Compared with the control group, a significant stimulation of biofilm formation and growth of *Arthrobacter* sp. (TB) was observed at concentrations of 25–50 μg ml^−1^ for diosmetin and 10–100 μg ml^−1^ for quercetin (Fig. 6B). Similarly, aromadendrin at a concentration of 50–100 μg ml^−1^ and eriodictyol at a concentration of 25–50 μg ml^−1^ significantly stimulated the biofilm formation and growth of *P. aeruginosa* (6a) (Fig. 6C). Furthermore, kaempferol at a concentration of 50–100 μg ml^−1^ exhibited significant promotion of biofilm formation and growth for *P. polymyxa* (5a) (Fig. 6D). However, the growth of the soilborne pathogen *I. destructans* was significantly inhibited by diosmetin, quercetin, and kaempferol at concentrations ranging from 0.1 to 100 μg ml^−1^ (Fig. 6E–G).

## Discussion

In this study we observed that plants exhibited modified secondary metabolite production, particularly flavonoid secretion in response to suboptimal low or high light stresses. These compounds recruit beneficial microorganisms and suppress plant pathogens. This microbial modification of soil ultimately leads to a reduction in soilborne diseases in subsequent crops. Regulating root metabolism to attract native beneficial microbes and establish a defensive rhizosphere microbial community offers a promising approach to combat soilborne diseases. These findings underscore the development of light stress-dependent disease suppression in the rhizosphere, which supports the formulation of a targeted crop canopy light adjustment strategy for effective management of soilborne diseases.

Light stress induces microbial modifications that contribute to the formation of disease-suppressive soil. Soilborne diseases primarily arise from the accumulation of pathogens, which is a consequence of an imbalance in the rhizosphere microbiome [31]. Typically, plants, especially susceptible host plants, inadvertently attract pathogens to the rhizosphere facilitating the development of soilborne diseases [32, 33]. However, plants have developed mechanisms to leverage associated microbiomes for defense against pathogens under biotic or abiotic stresses [34, 35]. We have previously shown that *P. notoginseng* is highly susceptible to severe root rot disease under field conditions with artificially consistent light, due to the accumulation of soilborne pathogens [28, 36, 37]. Furthermore, successful cultivation of *P. notoginseng* under forests with natural fluctuating light conditions has resulted in reduced soilborne diseases [30]. This study demonstrates that maintaining optimal consistent light conditions leads to the accumulation of soilborne pathogens and root rot. Conversely, suboptimal low and high light stresses alleviated root rot disease in *P. notoginseng* by enriching beneficial microbiota in the rhizosphere, which suppressed soilborne diseases and enhanced the plant’s resistance to light stress.

Previous studies have reported that microbes enriched by light stress can promote plant growth and combat pathogens [38–40]. We have found that both suboptimal low and high light stresses enrich beneficial microbes, thereby enhancing stress resistance. High light stress fosters more effective microbial assemblies that suppress disease compared with low light stress. With high light stress, populations of *Pseudomonas* and *Arthrobacter* are enriched, whereas low light stress predominantly enriches *Paenibacillus*. Culturable isolates from the rhizosphere soil, especially *P. aeruginosa* (6a), *Arthrobacter* sp. (TB), and *P. polymyxa* (5a), demonstrated antagonistic activities against *I. destructans*, effectively reducing root rot of *P. notoginseng* and also enhancing plant photosynthesis.

Root exudates play a crucial role in fostering mutualistic relationships between plants and microbes, as well as recruiting specific microbes in response to biotic and abiotic stresses [11, 41, 42]. In this study, we observed an enhancement in secondary metabolism, particularly in flavonoid synthesis and secretion, under both suboptimal low and high light stress conditions. High light stress led to a greater secretion of flavonoids, including diosmetin, quercetin, aromadendrin, eriodictyol, and kaempferol, compared with suboptimal low light stress. Many studies have demonstrated the involvement of flavonoids in helping plants cope with various biotic and abiotic stresses, as well as their multifaceted roles in plant–microbe interactions within the rhizosphere [43]. Kaempferol and quercetin, two flavonoids identified in our light-induced metabolites, have been shown to alleviate salt stress and light stress in plants [44, 45].

Previous studies have shown that the receptor-like kinase OsRLCK160 interacts with and phosphorylates the bZIP transcription factor OsbZIP48 to regulate flavonoid accumulation, aiding UV-B tolerance in rice [46]. Furthermore, it has been observed that natural variation in rice flavones is predominantly caused by OsUGT706D1 (flavonoid 7-*O*-glucosyltransferase) and OsUGT707A2 (flavonoid 5-*O*-glucosyltransferase), contributing to UV-B tolerance in the natural environment [47]. Further investigation is warranted to explore the mechanisms by which light stress influences the accumulation of flavonoids in *P. notoginseng*.

Importantly, we have found that flavonoids, including diosmetin, quercetin, and kaempferol, secreted by roots in response to suboptimal low and high light stress not only inhibited the growth of the pathogen *I. destructans*, but also promoted the growth of beneficial bacteria. Flavonoids are well-known polyphenolic secondary metabolites that have been widely reported to inhibit pathogen growth *in vivo* or *in planta* [48–50]. Moreover, root exudates not only serve as carbon and nitrogen sources for microbial growth but also function as signaling molecules that exert multiple effects on microorganisms in the rhizosphere [51]. Flavonoids, known to be stress-mediated compounds, have been found to aid in the recruitment of beneficial microbes [43, 52, 53]. Our data support the notion that light stress can enhance the synthesis and secretion of diosmetin, quercetin, aromadendrin, eriodictyol, and kaempferol, serving as carbon sources to stimulate the growth of beneficial microbes and as signaling molecules to promote biofilm formation.

While flavonoids are well known as phytoalexins that defend against pathogens through mechanisms such as membrane disruption [54], biofilm formation [55], inhibition of cell envelope synthesis [56], nucleic acid synthesis, the electron transport chain, and ATP synthesis [57], their role as promoters of microbial growth is less documented. High light stress enhances metabolism, leading to increased secretion of various flavonoid compounds, which in turn enrich beneficial microbiota and suppress soilborne pathogens. Overall, light stress modifies the rhizosphere microbiome by altering the presence of flavonoids, which can inhibit or promote microbial growth, thereby contributing to soil disease suppression.

Although flavonoids are known for their defensive properties, it is conceivable that microbes have evolved mechanisms to circumvent these defenses by adapting to specific flavonoids. For instance, the exogenous addition of isoflavones has been shown to induce the expression of nod-lacZ fusions in *Bradyrhizobium japonicum*. Quercetin-3-*O*-galactoside has been reported to stimulate the spore germination of *Glomus etunicatum* and *Glomus macrocarpum* in alfalfa seeds [58]. Additionally, flavonoids facilitate the movement of PGPR in the soil/root interface, acting as signal molecules [59]. This dual role underscores the need for further investigation to elucidate the mechanisms through which flavonoids enhance the growth of beneficial microbes while suppressing pathogens.

In addition to flavonoids, light stress also induces alterations in other secondary metabolites, including terpenes, phenolic acids, and lipids. Lipids have been shown to enhance the heat and drought tolerance of various plant species, such as tomatoes, wheat, and sesame seeds, through a reduction in unsaturation [60, 61]. Key lipids, such as oleic acid and linolenic acid, play a crucial role in maintaining plant growth homeostasis by acting as signaling mediators [62, 63]. Additionally, terpenes and lipids also serve protective functions in plants during periods of stress [64, 65], and the presence of pathogens often leads to an increased production of sesquiterpene phytoalexins [66].

Previous research has demonstrated that cucurbitacin, a triterpenoid compound found in melons, regulates rhizosphere microbiota and enhances plant disease resistance [67]. In our study, we confirmed the promoting effect of the triterpenoid compound oleanonic acid on the growth of *P. polymyxa*. These findings indicate that terpenoids, phenolic acids, and lipid substances may be involved in microbial reassembly under light stress treatments. These results suggest that both low and high intensities of light stress can enhance secondary metabolism, resulting in the inhibition of pathogens and the promotion of beneficial microbes, leading to the formation of a disease-suppressive soil.

Signals from aboveground light stress are integrated by underground beneficial microorganisms, influencing the plant’s response to biotic and abiotic stresses. The perception of these signals by plant leaves alters root exudation patterns, enabling the rhizosphere microbiome to aid in stress mitigation. Previous reports have indicated that signals from foliar pathogens or insect damage also alter root exudation, triggering beneficial underground microorganisms to enhance plant stress tolerance [13, 14, 20, 68].

In this study, we have found that changes in aboveground light intensity are integrated by beneficial underground microorganisms along the microbiome–root–stem axis, enhancing the plant’s defense against soilborne pathogens. This builds on previous findings that suboptimal light conditions employ the rhizosphere microbiome, integrating along a microbiota–root–shoot circuit to enhance plant growth [20]. Consequently, we propose that adjusting crop canopy light, due to its controllability, is an effective strategy for managing the suppressive capacity against soilborne diseases. However, light signals are very intricate in regulating plant growth and metabolism. Further investigation is needed to understand the underlying mechanisms by which foliar light stress signals influence root secretion and subsequently recruit rhizosphere microbes.

**Figure 7:**
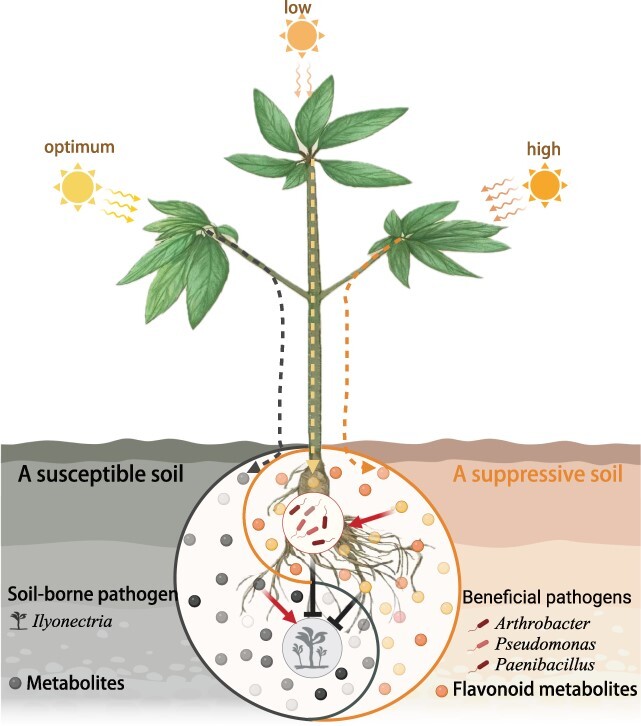
Schematic diagram of light stress-dependent elicitation of disease suppressive soils mediated by root-secreted flavonoids.

### Conclusions

Both suboptimal low and high light stresses can enhance the synthesis and secretion of secondary metabolites, thereby enriching beneficial microbiota in the rhizosphere to suppress soilborne diseases and cope with environmental stresses (Fig. 7). This unveils the light stress-dependent formation of disease-suppressive conditions in the rhizosphere, thus providing insights for developing a crop canopy fluctuating light adjustment strategy to effectively manage root rot disease in the field, promoting sustainable agriculture practices.

## Materials and methods

### Experimental design and sampling

*Panax notoginseng* was treated with optimal, suboptimal low and high light stresses. The experiments were conducted in a glasshouse and a forest located in Xundian County, Yunnan, China (103.13°E, 25.67°N; altitude 1960 m). The glasshouse soil had the following characteristics: pH 6.6, electrical conductivity 337.7 μS cm^−1^, organic matter 21 680 mg kg^−1^, available phosphorus 44.88 mg kg^−1^, available potassium 480.50 mg kg^−1^, and available nitrogen 84 mg kg^−1^. The underforest soil had the following characteristics: pH 5.17, electrical conductivity 458 μS cm^−1^, organic matter 47 830 mg kg^−1^, available phosphate 5.18 mg kg^−1^, available potassium 6.90 mg kg^−1^, and available nitrogen 172.38 mg kg^−1^. To create different light intensities in the glasshouse, a shade cover with a permeable black plastic net was utilized (Fig. 1). The light levels in the forest were selected based on the density of pine trees (Fig. 1). Light intensity was measured from 7 a.m. to 7 p.m. on a sunny day using a portable illuminance meter (TES-1339 R; Taishi, Taiwan, China). Three light intensities were set up in the glasshouse and the underforest setting corresponding to 5, 15, and 30% of full sunlight (~100 000 lux), which were defined as suboptimal low (L), optimal (M), and high (H) light intensities, respectively. In the forest experiment, each plot (2 m × 1.5 m) consisted of ~160 plants. In the glasshouse experiment, each shade covered an area of 2 m × 1.5 m × 1.5 m, containing 10 pots, each pot housing eight plants. Each treatment had six replicates in both experiments.

After 2 months, seedling survival and photosynthetic ability were assessed. Rhizosphere soil was collected and stored at −80°C for microbial analysis [37], while bulk soil was prepared for analysis of disease-suppressive ability. After rinsing with purified water, intact leaves and fibrous roots were collected and stored at −80°C for metabolic analysis.

### Seedling survival and photosynthesis

Seedling survival and chlorophyll fluorescence of the leaves were measured prior to sample collection. Chlorophyll fluorescence measurements were conducted using the IMAGING-PAM Chlorophyll Fluorometer (Heinz Walz, Effeltrich, Germany) following the methodology of previous studies [37, 69]. A total of 30 plants were measured in each treatment group. Measurements of the chlorophyll fluorescence ratio *F*_v_/*F*_m_, reflecting the photochemical maximum quantum efficiency of photosystem II (PSII), were performed between 9 a.m. and 11 a.m.

Diseased plants were sampled for pathogen isolation using a previously established method [70]. Briefly, freshly collected roots with lesions were surface-sterilized with 1% sodium hypochlorite for 5 min, rinsed with sterile water, and then cut into ~5-mm (in length) pieces. These samples were placed on potato dextrose agar (PDA) supplemented with 100 μg ml^−1^ chloramphenicol and incubated in the dark at 25°C for 7 days. Individual hyphal tips were then transferred to PDA plates for further development and purification. The isolated strains were identified by sequencing the internal transcribed spacer (ITS) region [36].

### Effects of light stress-conditioned soils on subsequent *P. notoginseng* growth

Bulk soil was obtained from both the underforest and glasshouse experiments after removing all plant materials. The experiment on subsequent *P. notoginseng* seedling emergence followed a methodology described previously [36]. Briefly, each bulk soil sample was divided equally into two parts. One portion was sterilized by autoclaving (St) at 121°C for 20 min, while the other portion remained untreated (U_St). These soils were then placed into pots, with eight disinfected *P. notoginseng* seeds sown in each pot. The pots were placed in a growth chamber configured to a photoperiod of 16 h light and 8 h dark, with a stable temperature maintained at 25 ± 2°C. The experimental design consisted of three replicates for each treatment, with six pots in each replicate. After 3 months assessments were conducted to measure seedling emergence and root rot incidence.

### Isolation and identification of potential beneficial microorganisms and pathogens

The effects of light stress on rhizosphere pathogenic fungi and beneficial bacteria were examined. Bacteria and fungi were isolated from rhizosphere soil samples following the method described by Luo *et al*. [14]. Firstly, 10 g of rhizosphere soil was vigorously mixed with 90 ml of sterilized water for 15 min. Subsequently, the mixture was serially diluted from 10^−1^ to 10^−7^. A sample of 100 μl was taken from each solution and spread onto plates containing nutrient agar and PDA, both amended with 100 μg ml^−1^ chloramphenicol. The plates were incubated at 25°C for 4–5 days. Individual colonies that emerged were subcultured on nutrient agar for bacteria or PDA for fungi in order to obtain pure cultures. These isolates were then identified by establishing a neighbor-joining tree as previously described [71].

### Microbial community analysis

Soil DNA was extracted using the Power Soil DNA isolation kit (Mo Bio Laboratories, Carlsbad, CA, USA), following the manufacturer’s instructions. For fungal communities, amplification of the ITS regions was carried out with primers ITS1F (CTTGGTCATTTAGAGGAAGTAA) and ITS2R (GCTGCGTTCTTCATCGATGC). Bacterial 16S rRNA genes were amplified with primers 308F (ACTCCTACGGGAGGCAGCAG) and 806R (GGACTACHVGGGTWTCTAAT) [72]. The PCR products were purified and sequenced using the Illumina MiSeq platform at Majorbio Bio-Pharma Technology (Shanghai, China).

The sequencing reads were quality-filtered using Trimmomatic before being merged with FLASH [73]. Subsequently, the high-quality sequences were grouped into operational taxonomic units (OTUs) with 97% similarity using UPARSE 7.1 [74]. The taxonomic classification of each representative sequence was performed using the RDP Classifier version 2.2 against the 16S rRNA gene database (e.g. Silva v138) with a confidence level of 0.7 [75].

In order to identify the significantly abundant bacterial and fungal taxa (from phylum to genus) across the various groups [linear discriminant analysis (LDA) score > 2, *P* < 0.05], we used the LDA effect size (LEfSe) method [76]. A correlation network was created to investigate the association between microbial biomarkers and seedling emergence of *P. notoginseng*. Correlations were considered statistically robust if Spearman’s correlation coefficient |*R*| > 0.5 and *P* < 0.05.

### Functions of beneficial bacteria isolated from rhizosphere soil

The antagonistic capacities of beneficial microorganisms against *I. destructans* were assessed using a dual culture experiment, adopting the approach given in a prior study [14]. In brief, a mycelial plug of the pathogen was co-cultured with a beneficial microbe on a PDA plate. Control plates were inoculated with only the pathogen on PDA. Each treatment consisted of four replicated plates, and all plates were incubated at 25°C for 5 days. The mycelial growth of the pathogen was assessed by measuring the colony’s semidiameter, and then the rate of growth inhibition was calculated.

To assess the impact of microorganisms on the survival rate of *P. notoginseng*, a pot experiment was conducted following the method of Luo *et al*. [14]. Sterilized soil was filled into pots, which were planted with eight plants each and inoculated with a proportional mixture of 50 ml of *I. destructans* (10^6^ CFU ml^-1^) along with one of the following: *P. aeruginosa* (6a) at OD_600_ of 0.5, *Arthrobacter* sp. (TB) at OD_600_ of 0.5, or *P. polymyxa* (5a) at (OD_600_ of 0.5. Control pots were treated with a proportional mixture of water and *I. destructans* (10^6^ CFU ml^-1^). Control pots received a mixture of water and *I. destructans* (10^6^ CFU ml^-1^). The pots were placed in a growth chamber set to a 16-h light/8-h dark photoperiod at a temperature of 25 ± 2°C. Each treatment consisted of three replicates, with five pots per replicate. After 2 months of growth, seedling survival was recorded.

### Metabolomic analysis of fibrous roots

Sample preparation and metabolite extraction were performed following the method described in Luo *et al*.’s study [14]. In brief, 50 mg of freeze-dried sample powder was extracted using 1.2 ml of 70% methanol. The sample was vortexed six times for 30 s each, with a 30-min interval between each vortexing step. The extracts were centrifuged at 12 000 rpm for 3 min and filtered using an SCAA-104 membrane with a 0.22-μm pore size (ANPEL, Shanghai, China).

The samples underwent analysis using the UPLC–ESI–MS/MS system (UPLC, ExionLC™ AD; MS, Applied Biosystems 4500 QTRAP). Analytical conditions for the UPLC analysis included an Agilent SB-C18 column (1.8 μm, 2.1 mm × 100 mm), with a mobile phase comprising 0.1% formic acid in water (solvent A) and 0.1% formic acid in acetonitrile (solvent B). Sample measurements were conducted with a gradient program starting at 95% A and 5% B. The program then transitioned to 5% A and 95% B over 9 min, remaining constant for 1 min. Following this, a composition of 95% A and 5.0% B was achieved in 1.1 min and maintained for 2.9 min. The flow velocity was set at 0.35 ml per minute, the column oven temperature at 40°C, and the injection volume at 4 μl. The effluent was directed to an ESI-triple quadrupole-linear ion trap (QTRAP)-MS. The stability and reliability of the experimental data were assessed using quality control (QC) samples. Triple quadrupole (QQQ) scans were performed using an API 6500 QTRAP LC/MS/MS System with an ESI turbo ion-spray interface, operating in positive ion mode and controlled by Analyst 1.6 software (AB Sciex). QC samples were created by combining equal quantities of all samples and evaluated every three samples to assess the repeatability of the MS results. Metabolite profiling was conducted by Wuhan Metware Biotechnology (Wuhan, China) utilizing a widely targeted metabolome approach (http://www.metware.cn/). Quantification of metabolites was performed using a multiple reaction monitoring method. Differentially accumulated metabolites (DAMs) were identified based on a variable importance of projection (VIP) value ≥1 and fold change ≥2 or fold change ≤0.5. A correlation network was established to explore the relationship between DAMs and biomarkers [77], with statistically robust defined as Spearman’s correlation coefficient |*R*| ≥ 0.6 and *P* ≤ 0.05.

### Impacts of flavonoids on beneficial bacteria and pathogenic fungi

To measure the impact of flavonoids on the growth of *P. aeruginosa* (6a), *Arthrobacter* sp. (TB), and *P. polymyxa* (5a), a previously published method was employed with some modifications [78]. Briefly, *P. aeruginosa* (6a), *Arthrobacter* sp. (TB), and *P. polymyxa* (5a) were cultured in nutrient agar liquid medium, and the cell density was adjusted by diluting them to a final OD_590_ of 0.4. Subsequently, 20 ml of fresh bacterial suspensions and 180 ml of nutrient agar were dispensed into 96-well microplates, resulting in final flavonoid (diosmetin, quercetin, aromadendrin, eriodictyol, and kaempferol) concentrations of 0.1, 1, 10, 25, 50, and 100 μg ml^−1^ in each well. The microplates were incubated at 28°C for 52 h, and the absorbance at 590 nm was measured using a Versa Max microplate reader (Molecular Devices, Sunnyvale, CA, USA) to generate bacterial growth curves (OD_590_ reads). Each concentration was tested in six replicates.

Biofilm formation of bacteria was assessed using a previously described method [79]. Briefly, the diluted culture was incubated anaerobically in 96-well microplates with varying concentrations of flavonoids (0.1, 1, 10, 25, 50, and 100 μg ml^−1^) for 52 h at 28°C. Following removal of the remaining bacterial culture from the wells, bacterial cells adhering to the well surfaces were gently washed twice with phosphate-buffered saline (1×) (PBS), air-dried, and subsequently stained with 200 μl of 0.1% (wt/vol) crystal violet for 15 min. After washing the wells four times with PBS to remove excess dye, the cell-bound dye was eluted using 200 μl of 99% methanol. Biofilm quantification was performed by measuring its optical density at 490 nm (OD_490_). Each treatment had 30 replications.

Diosmetin, quercetin, and kaempferol were amended into PDA at final concentrations of 0, 0.1, 1.0, 10, 25, 50, and 100 μg ml^−1^. Dimethyl sulfoxide (DMSO) was used as a solvent for the flavonoids, the final concentration being 1% in PDA. DMSO only was used as a control. Each treatment had six replicates. The plates were incubated at 25°C for 4 days, and the pathogen’s mycelial growth was measured by measuring the colony diameter perpendicularly.

### Statistical analysis

Statistical analysis was performed using the R package (version 3.3.1, https://cloud.majorbio.com/page/tools.html). One-way analysis of variance (ANOVA) and Duncan’s multiple-range test (α = 0.05) were employed for statistical analysis using SPSS 19.0 software (SPSS, USA). Correlation analysis was performed using the tools provided by OmicStudio at https://www.omicstudio.cn/home.

## Supplementary Material

Web_Material_uhae213

## Data Availability

The data supporting the findings of this study are available within the paper and its supplementary information files. The raw sequencing reads were deposited in the NCBI Sequence Read Archive (SRA) database. The underforest fungi and bacteria are denoted by PRJNA1074228 and RJNA1074125, respectively. The glasshouse fungi and bacteria are denoted by PRJNA1074256 and PRJNA1074252, respectively.
