# Supplementary material for: Light stress elicits soilborne disease suppression mediated by root-secreted flavonoids in Panax notoginseng
Source: Hortic Res. 2024 Jul 30;11(10):uhae213. doi: 10.1093/hr/uhae213 (PMC11462611; doi:10.1093/hr/uhae213)
Supplement: Web_Material_uhae213 [file web_material_uhae213.zip › Supplementary_figures.pdf]

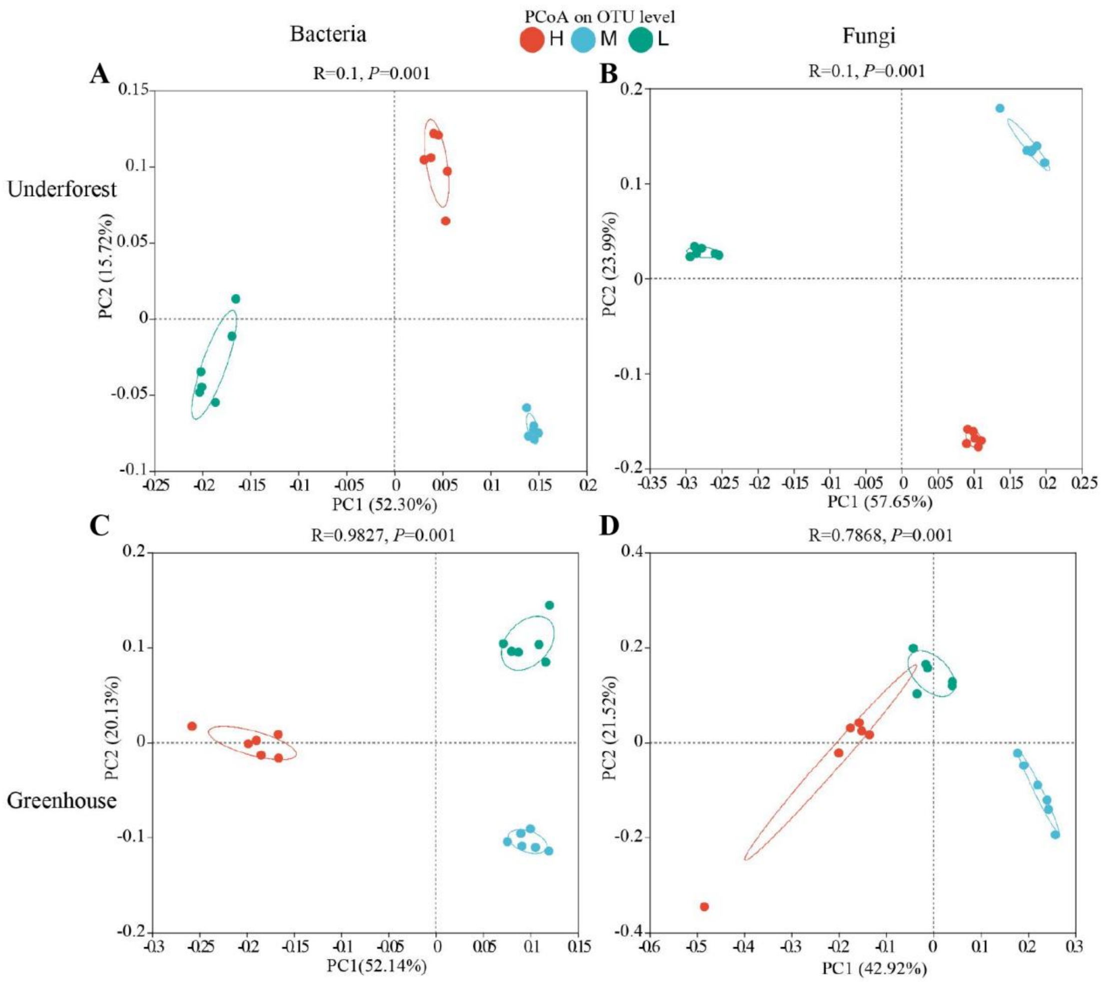

Supplementary Fig. S1. **PCoA analysis of rhizosphere microorganisms in the underforest and glasshouse. (A-B)** PCoA plots were generated to analyze the composition of rhizosphere bacteria **(A)** and fungi **(B)** in the growth of *P. notoginseng* under three different light intensity treatments in underforest. **(C-D)** PCoA plots were generated to analyze the composition of rhizosphere bacteria **(C)** and fungi **(D)** in the growth of *P. notoginseng* under three different light intensity treatments in glasshouse.

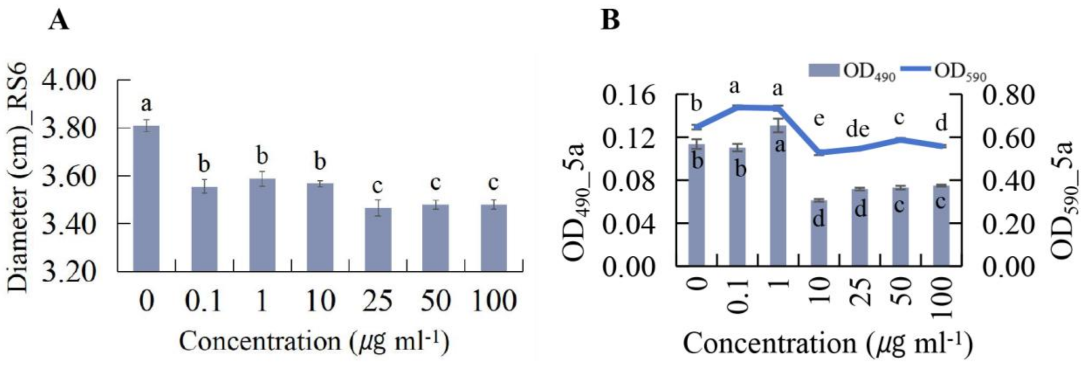

Supplementary Fig. S2. The impact of terpenes on the growth of fungi and bacteria.

(A-B) The influence of oleanonic acid on the growth of *I. destructans* **(A)** and the biofilm formation and growth of *P. polymyxa* (5a) **(B)** strain is evaluated.
